# Exosomes, their sources, and possible uses in cancer therapy in the era of personalized medicine

**DOI:** 10.1007/s00432-024-06066-w

**Published:** 2024-12-26

**Authors:** Tomas Zemanek, Lubos Danisovic, Andreas Nicodemou

**Affiliations:** 1 Institute of Medical Biology, Genetics and Clinical Genetics, Faculty of Medicine, Comenius University, Bratislava, Slovakia; 2 GAMMA - ZA s.r.o, Trencin, Slovakia

**Keywords:** Exosomes, Cancer, Immunotherapy, Tumor, Personalized medicine, Cell therapy

## Abstract

Despite significant advances in immunotherapy, its efficacy in solid tumors remains limited. Exosomes, a primary type of extracellular vesicles, can transport diverse intracellular molecules to nearby or distant cells and organs, facilitating numerous biological functions. Research has shown that exosomes have the dual ability to both activate and suppress the immune system. Their potential as anticancer vaccines arise from the capacity to carry antigens and major histocompatibility complex (MHC) molecules. Exosomes derived from blood, saliva, urine, and cerebrospinal fluid serve as promising biomarkers for cancer diagnosis and prognosis. Recent advancements in exosome-based therapy have highlighted its utility in drug delivery and immunotherapy. This review examines the composition and sources of exosomes within the immune microenvironment of solid tumors and delves into the mechanisms and pathways through which exosomes impact immunotherapy. We further explore the clinical potential of engineered exosomes and exosome vaccines in solid tumor immunotherapy. These insights may pave the way for exosome-based strategies in cancer diagnosis, treatment, and prognosis, enhancing the effectiveness of immunotherapy for solid tumors.

## Highlights

Exosome traits make them ideal for drug delivery and as targeted antitumor therapies.

Exosomes can mediate resistance to nearly all therapeutic drugs.

Exosomes are stable, immune-evasive, and penetrate barriers like the BBB easily.

CAR-Exo & NK-Exo penetrate tumors, showing great potential for solid cancer therapy.

## Introduction

Solid tumors have historically exhibited limited responsiveness to therapies effective in treating hematologic malignancies, primarily due to unique challenges within the tumor microenvironment (TME). In contrast, hematologic cancers generally respond better to immunotherapy, owing to distinct immune-tumor cell interactions in their microenvironments. The TME of solid tumors introduces several obstacles to treatment efficacy, including hypoxic conditions, nutrient deprivation, and acidity that collectively inhibit T-cell function and impede drug penetration. Additionally, structural components like the extracellular matrix (ECM), tumor vasculature, and cancer-associated fibroblasts create barriers that limit immune cell infiltration and reduce drug effectiveness (Sharma and Allison 2015; Demaria et al. 2019).

The TME comprises a complex ecosystem of tumor cells, immune cells (e.g., T cells, natural killer cells), blood vessels, and ECM. Within this environment, cellular communication occurs through cytokines, chemokines, and extracellular vesicles (EVs), such as exosomes, ectosomes, and apoptotic bodies. These lipid-based vesicles, containing nucleic acids, proteins, and lipids, facilitate cell-to-cell interactions and are involved in tumor progression and metastasis (Ren et al. 2018; Xiao and Yu 2021).

The primary EV subtypes - exosomes, ectosomes, and apoptotic bodies are categorized by their size, biogenesis, and function. However, overlaps in their protein compositions often occur due to variations in isolation and analysis techniques. (Borges et al. 2013; Yáñez-Mó et al. 2015). Notably, exosomes are particularly important in intercellular signaling within the TME. As stable, membrane-bound vesicles measuring 30–150 nm, they can transport bioactive molecules across cells, directly impacting tumor behavior and providing potential therapeutic insights (Cocucci and Meldolesi 2015). Exosome-related research is continuously updated, with the International Society for Extracellular Vesicles recently releasing the fourth edition of MISEV (Minimal Information for Studies of Extracellular Vesicles) in 2023, offering standardized guidelines for EV research (Welsh et al. 2024).

## Discovery, origin, and purpose of exosomes

Exosomes were first discovered by Wolf in (1967) and later extensively studied by Johnstone et al. in (1987), who observed them during the maturation of sheep reticulocytes. Early research highlighted their crucial role in transporting membrane proteins and facilitating various cellular functions (Johnstone et al. 1991). Subsequent studies identified exosomes in B lymphocytes, uncovering their involvement in immune regulation, including T-cell activation and tumor suppression. Over time, exosomes have been found in numerous cell types, such as mesenchymal stem cells, dendritic cells, epithelial cells, and tumor cells (Yu et al. 2014; Shaban et al. 2022).

**Fig. 1:**
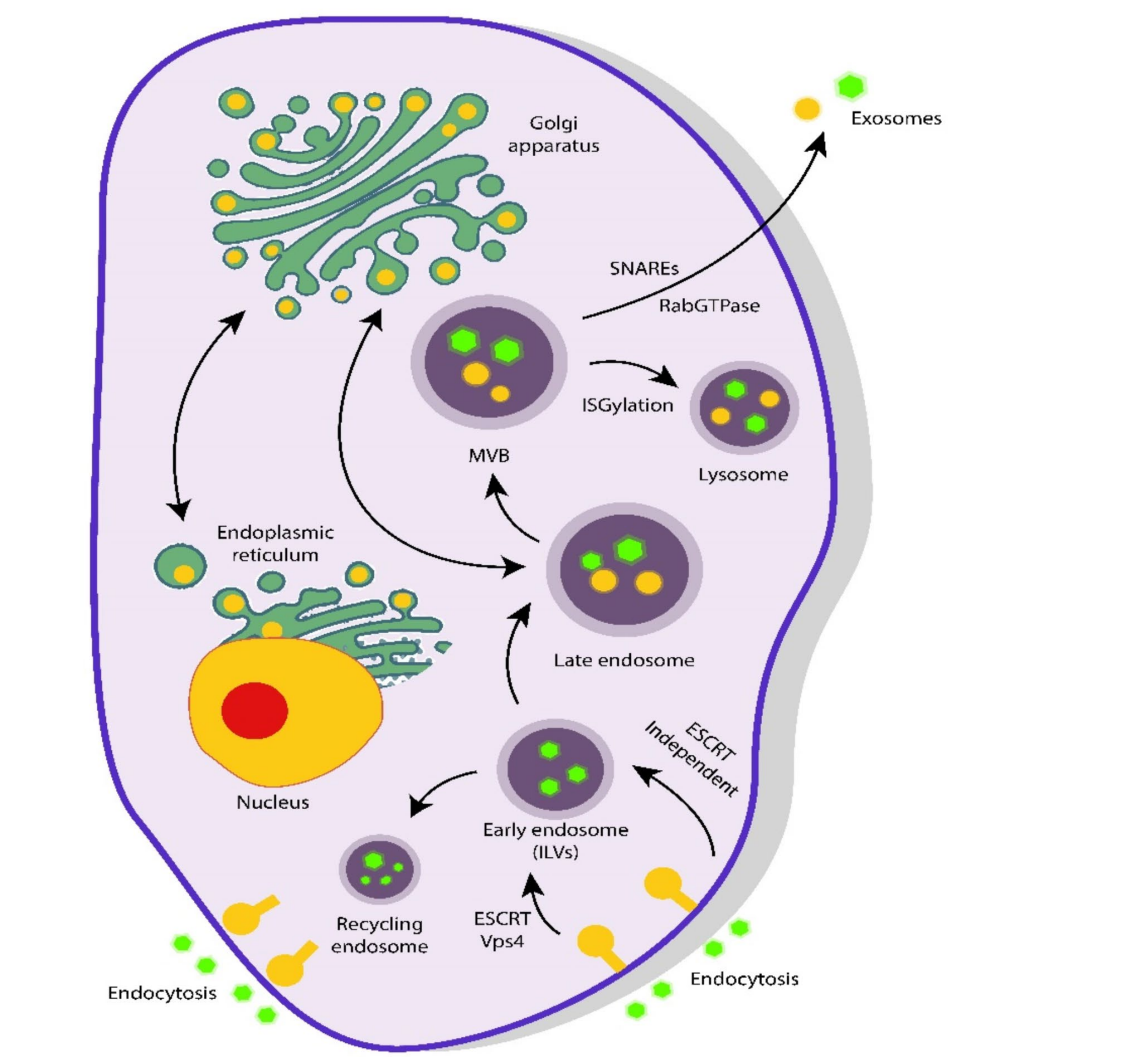
The creation of exosomes in donor cells

Exosome formation starts with endocytosis, during which intraluminal vesicles (ILVs) are generated within endosomes. These endosomes develop into multivesicular bodies (MVBs), which either undergo degradation or fuse with the plasma membrane to release exosomes into the extracellular environment, as illustrated in Fig. 1. The release process is regulated by proteins such as *Rab* and *Ral*, as the decline in their levels lead to decreased exosome secretion. Additionally, exosomes respond to inflammation and stress, underscoring their involvement in immune responses (Ostrowski et al. 2010; Hyenne et al. 2015).

Exosomes influence target cells through various mechanisms like binding to cell surface receptors, fusing with target cells to deliver specific factors, or releasing intracellular substances that activate target cell receptors (Welsh et al. 2024). They play critical roles in processes like immune regulation, tissue repair, and cancer progression by transporting biological signals that alter target cell functions and gene expression. Consequently, exosomes hold significant potential in immunotherapy, regenerative medicine, and cancer treatment (Elahi et al. 2020).

## Substances carried by exosomes

According to database records, exosomes in cells contain thousands of proteins, along with numerous mRNAs, miRNAs, and lipids (Keerthikumar et al. 2016). The protein composition of exosomes varies based on their cell of origin and includes diverse types, such as cytoplasmic enzymes, fusion proteins (e.g., integrin), membrane transport proteins (e.g., GTPase), molecular chaperones like HSP60, and structural proteins such as actin. Exosomes also carry proteins involved in signal transduction, including G proteins and protein kinases, as well as tissue-specific proteins like MHC-I. Rich in lipids, they contain glycerol, phospholipids, cholesterol, and glycosylceramides. Additionally, exosomes transport functional RNAs (miRNAs, noncoding RNAs, and mRNAs) and DNA, being involved in tumor development through interactions within the tumor microenvironment (TME). Both, immune cells such as T cells, B cells, or macrophages as well as tumor cells release exosomes. These often carry donor cell antigens, nucleic acids, and proteins that interact with receptors on target cells, modulating immune responses. Research indicates that exosomes from immune and cancer cells have dual roles, contributing to both tumor progression and suppression, underscoring their complex involvement in cancer development and immune regulation. (Théry et al. 2002, 2009).

## Types of exosomes and their antitumor activity

### Immune cells derived exosomes

#### T cells-derived exosomes

T cells, pivotal players in antitumor immunity, are the most prevalent tumor-infiltrating lymphocytes in the TME (Chow et al. 2022). Exosomes derived from T cells also contribute to immune activation. For instance, exosomes from expanded Vδ2-T cells have been shown to induce apoptosis in Epstein-Barr virus (EBV)-associated gastric carcinoma cells, thereby suppressing tumor growth in Rag2^−/−γc−/−^ mice (Wang et al. 2021). Similarly, exosomes from CD45RO^−^CD8^+^ T cells containing miR-765 were found to partially inhibit estrogen-driven endometrial cancer progression by modulating epithelial-mesenchymal transition (EMT) (Zhou et al. 2021). CD4^+^ T cells are capable of activating CD8^+^ T cells to differentiate into cytotoxic T lymphocytes (CTLs), thus fine tuning CTLs antitumor responses. Various miRNAs found in exosomes derived from CD4^+^ T cells enhanced CD8^+^ T cell mediated antitumor responses in melanoma under IL-2 stimulation (Shin et al. 2022).

Regulatory T cells (Tregs), a subgroup of immunosuppressive CD4^+^ T cells, are crucial for maintaining immune homeostasis and self-tolerance. However, they can suppress immune surveillance in cancer, hindering antitumor responses and promoting tumor progression (Tay et al. 2023). In a B16F10 mouse melanoma model, activated CD8^+^CD25^+^ Tregs were found to release exosomes containing IL-10 and TGF-β, which modulate lymphocyte activity and suppress CTLs responses (Xie et al. 2013). Meanwhile, T cells are pivotal for the success of modern cancer immunotherapies. Chimeric antigen receptor T cells (CAR-T), engineered to express chimeric antigen receptors, demonstrate precise targeting capabilities. These cells have been reported to release CAR-containing exosomes (CAR-Exo) that exhibit antitumor activity against several solid tumors, such as breast, lung, and ovarian cancers (Fu et al. 2019).

#### B cells-derived exosomes

B cells, originating from multipotent stem cells in the bone marrow, have the unique capacity to produce antibodies. Upon antigen stimulation, they proliferate and differentiate into plasma cells, which secrete specific antibodies into the bloodstream. These antibodies mediate their effects through mechanisms such as antibody dependent cell mediated cytotoxicity (ADCC) (Laumont et al. 2022). Additionally, as antigen presenting cells, B cells can directly activate T cells and macrophages, playing a significant role in TME.

The function of B cell derived exosomes is being increasingly understood. CD19^+^ exosomes released by B cells are enriched in CD39 and CD73 molecules, which hydrolyze ATP from apoptotic tumor cells into adenosine. This process suppresses CD8^+^ T cell activation during chemotherapy, ultimately reducing its antitumor effectiveness. Elevated levels of CD19^+^ exosomes have been observed in the serum of cancer patients across various stages compared to healthy individuals, with lower CD19^+^ exosome levels correlating with better chemotherapy outcomes. Moreover, studies indicate that the TME can upregulate HIF-1α protein levels in B cells, enhancing exosome secretion. These findings emphasize the diagnostic potential of serum CD19^+^ exosome levels for monitoring tumor progression and assessing chemotherapy efficacy (Zhang et al. 2019a).

#### NKs-derived exosomes

Natural killer (NK) cells play a vital role in innate immune surveillance and the TME. They secrete various cytokines and chemokines, recruit other immune cells, and enhance adaptive immune responses by T and B cells, in addition to directly killing tumor cells. NK cell activation through IgG antibodies induces ADCC, contributing to immune regulation and aiding in the management of autoimmune diseases, though it rarely triggers autoimmunity (Campbell and Hasegawa 2013; Maskalenko et al. 2022).

EVs secreted by NK cells carry signature NK markers and cytotoxic proteins, such as granzyme A and B, granulysin, and perforin, which mediate antitumor effects against solid tumors, such as neuroblastoma, breast cancer, and non-small cell lung cancer (NSCLC) (Lugini et al. 2012; Jong et al. 2017; Wu et al. 2019). Additionally, cytokines like interleukin-15 (IL-15) can enhance the antitumor potency of NK cell derived EVs against cancers such as thyroid or breast, and glioblastoma (Zhu et al. 2019). These exosomes may also carry tumor suppressive microRNAs (miRNAs) that inhibit neuroblastoma progression and counteract TGFβ1 dependent immune escape (Neviani et al. 2019). Furthermore, exosomes derived from expanded NK cells (eNK-Exo) have demonstrated antitumor activity against ovarian cancer cells, both alone and when loaded with cisplatin, as transcriptomic analyses suggest that eNK-Exo enhances NK cell mediated antitumor responses (Luo et al. 2023).

#### Dendritic cells-derived exosomes

Dendritic cells (DCs) are pivotal in antigen uptake, processing, and presentation to T cells, thereby driving antitumor immune responses (Wakim and Bevan 2011). Exosomes derived from α-fetoprotein (AFP) expressing DCs have been shown to remodel the TME and inhibit tumor progression in mouse models of ectopic, orthotopic, and carcinogen induced hepatocellular carcinoma (Lu et al. 2017).

Research indicates that DC derived exosomes (Dex) loaded with patient specific neoantigens effectively suppress tumor growth, prevent lung metastasis, and improve survival in B16F10 melanoma and MC-38 mouse models (Li et al. 2023). Similarly, cancer specific transcription induced chimeric RNA loaded Dex have demonstrated the ability to enhance CD8^+^ T cell mediated antitumor immunity and suppress tumor progression in esophageal cancer (Xiong et al. 2022). Additionally, Dex have shown promise in clinical applications. A phase II clinical trial (NCT01159288) demonstrated that Dex improved progression free survival in patients with advanced NSCLC by enhancing NK cell mediated antitumor immunity (Besse et al. 2016.

#### MDSCs-derived exosomes

Myeloid-derived suppressor cells (MDSCs) play critical roles in pathogenic and inflammatory immune responses. Renowned for their potent immunosuppressive abilities, MDSCs can dampen immune activity, modulate inflammation, and contribute to wound healing. However, tumor cells can disrupt bone marrow processes, leading to abnormal progenitor cell differentiation and MDSC accumulation, which undermines the efficacy of antitumor immunotherapy. Numerous studies have established the immunosuppressive function of MDSCs within the TME and their link to diminished antitumor immune responses (Veglia et al. 2021). For instance, in a breast tumor mouse model, doxorubicin treatment induced the proliferation of IL-13R^+^miR-126a^+^ MDSCs. Exosomes derived from these cells facilitated lung metastasis by promoting IL-13^+^ Th2 cell proliferation and enhancing tumor angiogenesis (Deng et al. 2017). Similarly, MDSC derived exosomes containing S100A9 protein have been shown to support colorectal cancer progression (Wang et al. 2019c). Another study revealed that tumor derived exosomal miR-9 and miR-181a promoted the infiltration and expansion of early stage MDSCs in breast cancer, suppressing T-cell immunity and driving tumor growth and immune evasion (Jiang et al. 2020).

#### Mast cells-derived exosomes

Mast cells (MCs), myeloid cells found in connective tissues, are rich in granules containing inflammatory mediators such as histamine. While they are traditionally linked to allergic and autoimmune diseases, MCs also significantly influence tumor cells and the TME. Acting as coordinators of antitumor immunity and regulators of the tumor matrix, MCs can exhibit either protumor or antitumor effects based on their location relative to the tumor and their interactions within the TME. These dual roles make MCs a compelling target for cancer immunotherapy (Lichterman and Reddy 2021).

Mast cell-derived exosomes (MC-Exo) can transfer oncogenic proteins to tumor cells, enhancing tumor progression through ligand-receptor interactions that activate signaling pathways. For instance, exosomes from the human mast cell line HMC-1 were efficiently taken up by the lung epithelial cell line A549. These exosomes carried the KIT protein, which activated KIT-SCF signaling, upregulated cyclin D1 expression, and promoted A549 cell proliferation (Xiao et al. 2014).

### MSCs-derived exosomes

Mesenchymal stem cells (MSCs) are multipotent stromal cells found in various tissues, including bone marrow, adipose tissue, umbilical cord, and dental pulp. They are characterized by their ease of cultivation and expansion in vitro, low immunogenicity, immunomodulatory properties, and natural migration to inflammation or tumor sites (Bianco et al. 2013). These features make MSCs highly valuable in clinical studies on inflammatory and tumor-related diseases. Under resting or stress conditions, MSCs produce large amounts of exosomes, which are essential for intercellular communication and share functional similarities with MSCs. MSC-derived exosomes (MSC-Exo) have complex dual roles in tumor biology, influencing angiogenesis, invasion, growth, metastasis, and drug resistance. Their effects can either promote or inhibit tumor progression, depending on the specific signaling pathways they activate and their influence on tumor development through protein molecule interactions (Weng et al. 2021; Lin et al. 2022b).

For example, bone marrow MSC-derived exosomes (BMSC-Exo) have been shown to disrupt DNA synthesis by inhibiting S-phase arrest, proliferation, and colony formation in NSCLC cells, thereby indirectly suppressing tumor growth (Liang et al. 2020). On the other hand, MSC-Exo can promote tumor invasion and metastasis by delivering specific miRNAs, such as miR-208a and miR-1587, which enhance the synthesis of proteins involved in these processes (Figueroa et al. 2017; Qin et al. 2020). Additionally, MSC-Exo can facilitate tumor progression by transferring cytokines like IL-6 to target cells (So et al. 2015).

Recent studies also reveal that MSC-Exo can upregulate proliferating cell nuclear antigen (PCNA), activate the ERK1/2 signaling pathway, and accelerate angiogenesis, thereby supporting tumor development (Zhu et al. 2012). Conversely, MSC-Exo have been found to inhibit angiogenesis by downregulating vascular endothelial growth factor (VEGF) through specific miRNAs, offering potential therapeutic benefits in cancer treatment (Pakravan et al. 2017).

### Tumor derived exosomes (TDEs)

**Fig. 2:**
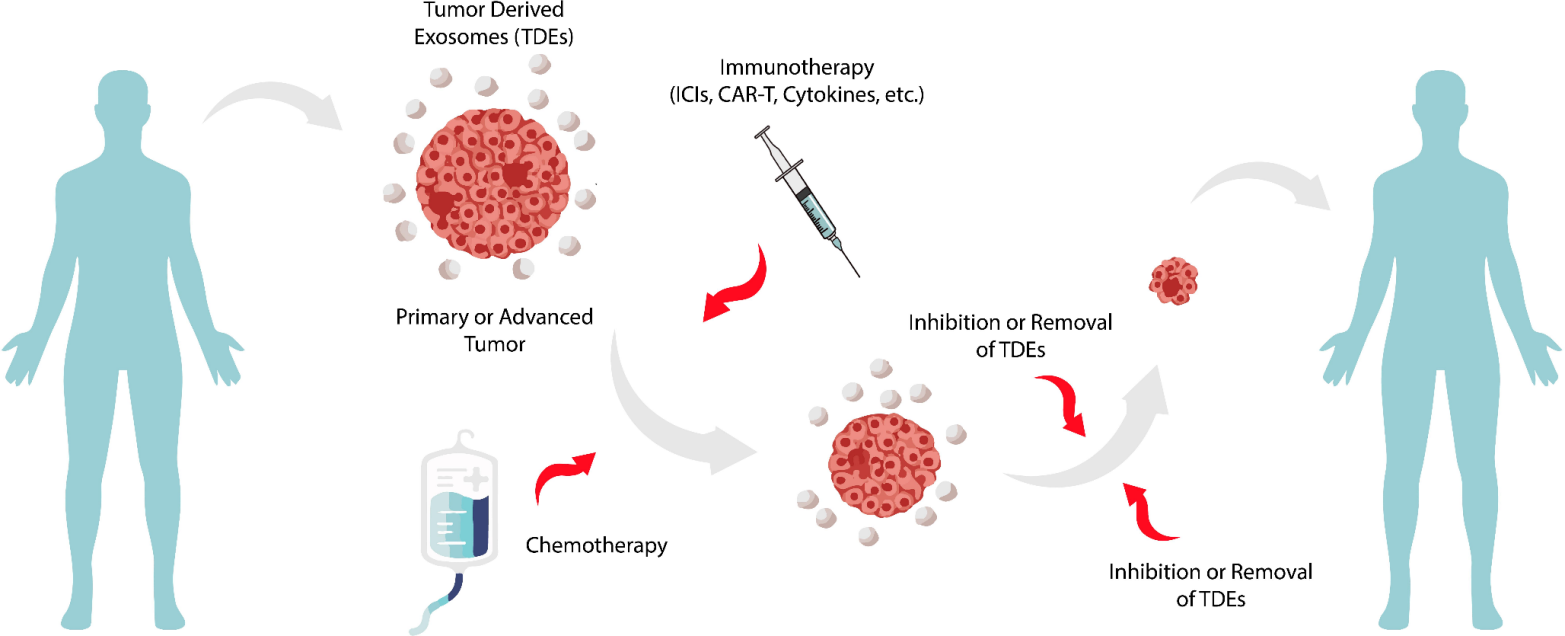
Tumor derived exosomes (TDEs) in solid cancer treatment

Tumor cells produce large quantities of exosomes, known as tumor-derived exosomes (TDEs), which transfer oncogenic material to neighboring cells, fostering a microenvironment favorable for tumor growth and metastasis. TDEs carry specific antigens, genes, and secretions from their parent cells, enabling them to interact with immune cells, promote the secretion of anti-inflammatory factors, and alter cell states to activate downstream immune responses (Zhang and Yu 2019b). However, TDEs can also suppress immune responses by leveraging signaling molecules, significantly reducing immunity, particularly in advanced stage cancer patients (Hosseini et al. 2021). The role of TDEs in solid tumor treatment is illustrated in Fig. 2.

TDEs from primary tumors recruit tissue resident interstitial macrophages (IM) to premetastatic niches, where they promote lactic acid secretion and upregulate immunosuppressive programmed death ligand 1 (PD-L1), creating an immunosuppressive environment conducive to solid tumor metastasis (Morrissey et al. 2021). For example, colorectal cancer (CRC) exosomes containing miR-424 disrupt T cell costimulatory signaling, reducing CD28 and CD80 expression in T cells and DCs. This impairment prevents full T cell activation and leads to resistance to immunotherapy in CRC patients (Zhao et al. 2021). Similarly, in NSCLC, silencing the ORAI1 channel in tumor cells decreased PD-L1 exosome secretion, enhanced CD8^+^ T cell infiltration, and inhibited tumor progression (Chen et al. 2022).

In hepatocellular carcinoma (HCC), the competitive binding of high-mobility group box 1 (HMGB1) and RICTOR in the mTOR pathway to miR-200 family members, such as miR-429, supports tumor self-renewal, tumorigenesis, and glutamine metabolism. This process generates PD-L1^+^ exosomes, which diminish the efficacy of anti-PD-L1 immunotherapy (Wei et al. 2021). Similarly, in oral cancer, the Lon protease regulates mitochondrial DNA (mtDNA) metabolism and mitochondrial reactive oxygen species (ROS) production, inducing the secretion of mitochondrial DNA and PD-L1 containing exosomes via the STING-IFN signaling pathway. These exosomes drive M2 macrophage secretion of immunosuppressive cytokines, impairing innate and CD8^+^ T cell immunity in the tumor microenvironment (Cheng et al. 2020).

In melanoma patients, serum derived exosomes expressing CD73 suppress interferon gamma (IFN-γ) production, impairing T cell function. Patients responding to anti-PD-1 therapies exhibit increased serum exosomal PD-L1 during treatment, while non-responders show elevated CD73 levels early in treatment, indicating that CD73 may predict poor therapeutic response (Turiello et al. 2022). Additionally, Hodgkin and Reed-Sternberg (HRS) cells phosphorylated at S345 via ERK signaling exhibit PD-L1 enrichment in exosomes, which bind to the extracellular matrix (ECM) and inhibit CD8^+^ T cell infiltration (Guan et al. 2022). Further, exosomes from triple-negative breast cancer (TNBC) cells carrying active TGF-β type II receptor (TβRII) stimulate TGF-β signaling in CD8^+^ T cells, inducing T cell exhaustion and reducing immunotherapy efficacy (Xie et al. 2022). Similarly, TDEs from metformin treated human ovarian cancer cells have been shown to increase exosome biogenesis, potentially mediating resistance to treatment by modifying the TME and facilitating intercellular communication (Abbasi et al. 2024).

### TAMs-derived exosomes

Tumor-associated macrophages (TAMs) play a pivotal role in driving tumor progression within the TME (Vitale et al. 2019). Macrophages are typically classified into two types: M1 and M2 (Murray 2017). M1 macrophages, activated by cytokines such as IFN-γ, produce proinflammatory and immunostimulatory cytokines, including IL-12 and TNF-α. In contrast, TAMs generally resemble M2 macrophages, which are activated by Th2 cytokines. TAMs contribute to tumor progression by facilitating cell invasion, proliferation, and metastasis, promoting tumor angiogenesis, and suppressing antitumor immune responses (DeNardo and Ruffell, 2019). These characteristics have made TAMs a focus of interest as potential therapeutic targets.

TAMs interact with tumor cells through exosome secretion (Zheng et al. 2018). While TAM derived exosomes are generally immunosuppressive, but they also possess the capacity to stimulate antitumor immunity. Proteomic analyses of exosomes from macrophages in mouse colon tumors have shown that TAM derived exosomes express molecular markers associated with Th1/M1 cells, enhancing immune responses, driving inflammation, and correlating with improved patient outcomes. These exosomes contain bioactive lipids and enzymes that may modulate proinflammatory signaling in cancer cells (Cianciaruso et al. 2019). Exosomes from proinflammatory M1 macrophages can also activate the NF-κB pathway in M0 macrophages, driving their differentiation into the M1 phenotype. This transition induces the secretion of proinflammatory cytokines such as IL-6, IL-12, and TNF-α, creating a localized inflammatory environment. In a mouse breast tumor model, these exosomes were shown to trigger tumor cell apoptosis and suppress tumor growth (Wang et al. 2019a).

TAMs and their exosomes are instrumental in establishing the premetastatic niche (Teng et al. 2016). For example, low serum levels of miR-208b in CRC patients are associated with longer progression free survival. CRC cells secrete miR-208b via exosomes to promote Treg expansion, directly inhibiting PDCD4 expression in CD4^+^ T cells, facilitating Treg differentiation, and contributing to immunosuppression in CRC mouse models (Ning et al. 2021). Additionally, CRC derived exosomal miR-934 has been shown to promote liver metastasis by inducing M2 macrophage differentiation and supporting the development of the premetastatic niche (Zhao et al. 2020).

### CAFs-derived exosomes

Cancer-associated fibroblasts (CAFs), key stromal cells within the TME, are defined by distinct biological markers and play a pivotal role in tumor progression. CAFs contribute to cancer development by inhibiting apoptosis and promoting cell proliferation, migration, invasion, drug resistance, and immune evasion. These effects are achieved through mechanisms such as preserving tumor cell stemness, enhancing angiogenesis, remodeling the extracellular matrix, suppressing immune responses, and regulating metabolic pathways. Acting as intermediaries between tumors and the TME, CAFs mediate interactions that foster tumor growth, making them valuable targets for therapeutic strategies (Li et al. 2021).

Research has demonstrated that miR-500a-5p, highly expressed in CAF derived exosomes, is transferred to breast cancer cells, where it promotes proliferation and metastasis (Chen et al. 2021). Similarly, CAF derived exosomes containing the autophagy related protein GPR64 have been shown to drive invasion and metastasis when taken up by breast cancer cells, highlighting their role in facilitating tumor progression via the TME (Xi et al. 2021). In hypoxic breast cancer, CAF derived exosomal circHIF1A is upregulated, enhancing tumorigenesis and cancer cell stemness (Zhan et al. 2021).

In colorectal cancer, CAF derived exosomal miR-92a-3p suppresses mitochondrial apoptosis while promoting chemotherapy resistance, stemness, metastasis, and EMT (Hu et al. 2019). Conversely, exosomal DACT3-AS1 from CAFs has been shown to reduce invasion, migration, and proliferation in gastric cancer cells, while improving chemotherapy sensitivity (Qu et al. 2023). In head and neck cancer, CAF derived exosomes with low levels of miR-3188 have been observed to inhibit tumor growth by targeting BCL2 (Wang et al. 2019b). Meanwhile, in oral squamous cell carcinoma, CAF derived exosomal miR-34a-5p promotes EMT mediated metastasis (Li et al. 2018).

## Manufacturing exosomes for immunotherapy in solid cancers

Exosomes, being highly stable and biocompatible molecules, serve as efficient carriers for a wide range of bioactive substances, including nanomaterials, proteins, and nucleic acids. They play a crucial role in intercellular communication (Cocucci and Meldolesi 2015). Research has shown that natural exosomes derived from tumor or immune cells can trigger antitumor responses, making them promising candidates for cancer vaccines (Wu et al. 2023). However, the clinical application of natural exosomes faces several challenges. These include their sequestration in non-target tissues like the liver and lungs, which hampers in vivo targeting, and their complexity and heterogeneity, which complicate production and quality control, ultimately limiting therapeutic effectiveness (Liu et al. 2022b).

To address these issues, engineered exosomes have emerged as an advanced alternative in exosome-based therapies (Ahmadi et al. 2022). Compared to natural exosomes, engineered versions offer advantages such as enhanced stability, improved intracellular delivery, extended circulation time, superior tumor targeting, controlled drug release, and increased tumor site accumulation. These features collectively enhance the safety, specificity, and efficacy of these therapies (Zhang et al. 2021). Engineered exosomes achieve their targeted functionality through methods like biological fusion or chemical modification (Liang et al. 2021). For instance, researchers have created exosomes by transfecting T cells with GFP-PD-1 lentivirus, leading to PD-1 expressing exosomes capable of neutralizing PD-L1 and reinvigorating CD8^+^ tumor infiltrating T cells in melanoma models (Li et al. 2022). Similarly, exosomes derived from engineered Jurkat T cells expressing IL-2 on their surface were shown to reprogram miRNA levels, suppress PD-L1 expression in melanoma cells, and inhibit tumor growth in immunocompetent mouse models (Jung et al. 2022).

**Fig. 3:**
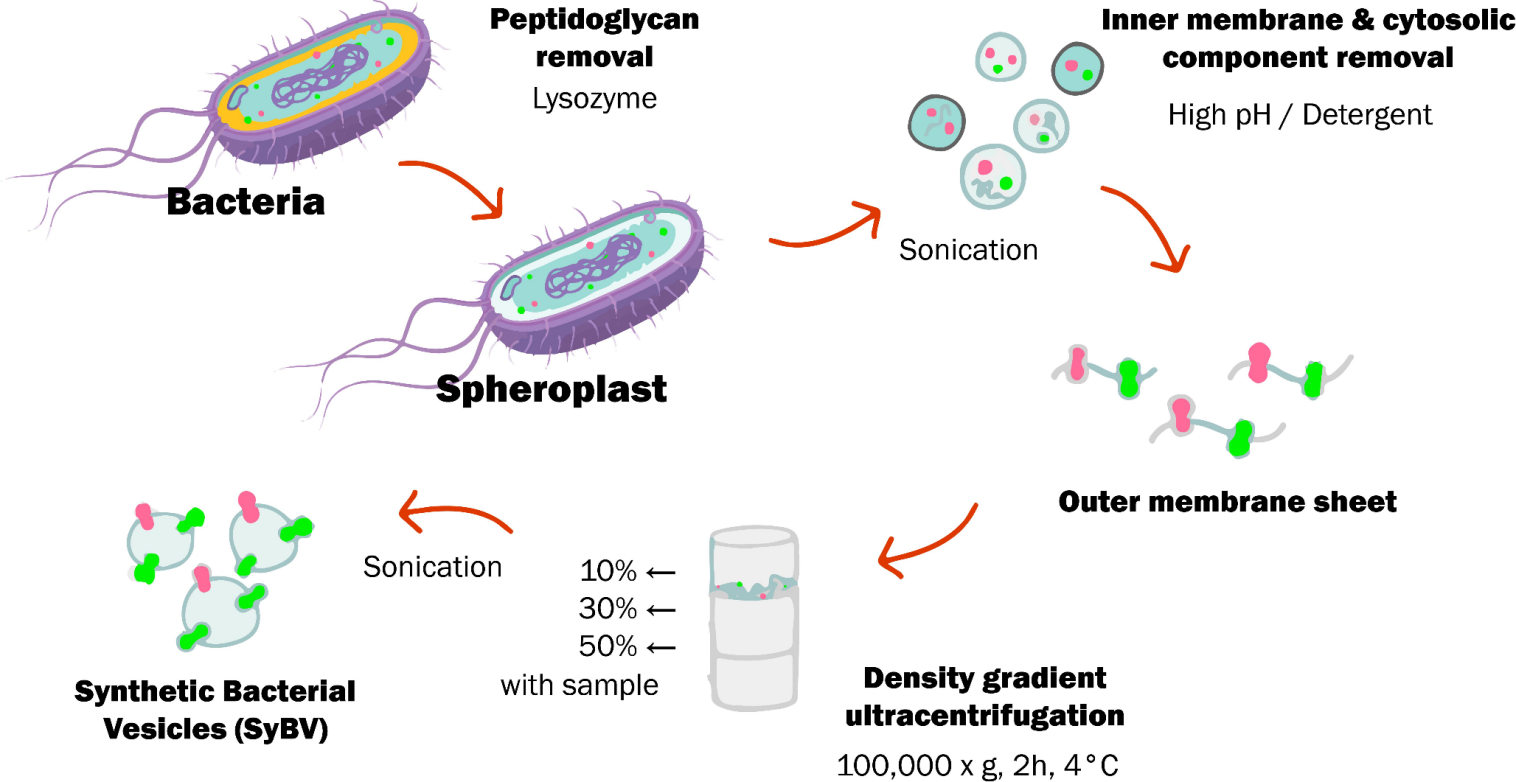
Synthetic bacterial vesicles (SyBV) production

In addition to exosomes, lipid nanoparticles such as outer membrane vesicles (OMVs), liposomes, and tumor cell membrane vesicles are being widely employed in tumor drug delivery systems (Chen et al. 2020). OMVs, secreted by gram-negative bacteria, contain outer membrane proteins, lipopolysaccharides, nucleotides, and lipids that can activate innate immunity via pathogen associated molecular patterns and antigens (Carvalho et al. 2019). Recent advances include the development of Synthetic Bacterial Vesicles (SyBV), engineered from bacterial exosomes treated with lysozyme and high pH, which is depicted in Fig. 3. Moreover, SyBV has also demonstrated the ability to avoid systemic proinflammatory responses in mice. When combined with melanoma derived exosomes, SyBV immunization promoted tumor regression in mouse models by balancing antibody production and supporting Th1 type T cell immunity (Park et al. 2021).

Synthetic biology has further enabled the engineering of bacterial outer membranes to present tumor neoantigens on OMV surfaces. One innovative system combined granulocyte-macrophage colony-stimulating factor (GM-CSF) with biomimetic bacterial derived vesicles in a hydrogel, allowing continuous GM-CSF release, recruitment of dendritic cells, and stimulation of T cell responses (Meng et al. 2022). Furthermore, tumor antigens were successfuly fused onto OMVs, which leveraged their natural interaction with the immune system to cross the intestinal barrier, where they were processed by antigen-presenting cells in the lamina propria. This approach generated robust antitumor immunity and immune memory in mouse models of metastatic melanoma and colon tumors (Yue et al. 2022).

Tumor immunotherapy, which enlists the immune system to recognize and destroy cancer cells, holds great promise as a treatment strategy. Despite this, the immunosuppressive TME remains a significant barrier. A novel dual-targeting exosome approach has been developed to address this challenge by leveraging the homing properties of TDEs and the immune activation capabilities of immune cell derived exosomes. Another innovative strategy involved introducing isolated tumor cell nuclei into activated macrophages to create hybrid cells, which were then used to produce reprogrammed macrophage derived tumor chimeric exosomes. These exosomes, loaded with immune components such as cytokines, co-stimulatory molecules, and MHC-I molecules, naturally targeted lymph nodes and accumulated in tumors. This method demonstrated significant inhibition of primary, recurrent, and metastatic tumor progression in models of melanoma and breast cancer (Wang et al. 2021).

## Exosome vaccines in solid cancers

Tumor immunotherapy includes various approaches such as tumor vaccines, oncolytic viruses, CAR-T cells, and other cellular therapies. Among these, tumor vaccines stand out for their unique ability to induce long term immunological memory, activate immunity, and provide synergistic therapeutic effects. Dendritic cell (DC) vaccines, in particular, exhibit strong antitumor activity due to their exceptional antigen presenting capabilities, effectively triggering antigen specific immune responses. However, cellular vaccines face limitations such as restricted cell availability, limited proliferation, challenges in production and storage, and complex quality control requirements (Lin et al. 2022a).

Exosomal vaccines, on the other hand, offer several advantages over traditional cellular vaccines, including a stable structure, well defined composition, ease of storage, enhanced immune specificity, and scalability for large scale production. For instance, researchers have engineered an exosomal vaccine called HELA Exos, which integrates a TLR3 agonist and an immunogenic cell death (ICD) inducer. This vaccine activates DCs in situ and induces ICD specifically in breast cancer cells, demonstrating significant tumor inhibition in xenograft mouse models with low immunogenicity TNBC and human derived tumor organoids (Huang et al. 2022).

Using biomimetic synthesis, another innovative platform, ASPIRE (Antigen Self-Presentation and Immunosuppression Reversal), was developed from engineered DC derived exosomes. ASPIRE employs DC membranes as natural signaling vectors to deliver multiple costimulatory signals, enabling robust multidimensional antitumor immune activation. This platform incorporates CD80/CD86 and an anti-PD-1 antibody, effectively reversing immune suppression and restoring T cell functions in models of Lewis lung carcinoma and MC-38 colon carcinoma (Liu et al. 2022a). Similarly, a chimeric RNA exosome vaccine that utilizes A-Pas as a novel antigen, combined with Dex, has shown promising results in inhibiting tumor growth and extending survival in esophageal cancer mouse models by remodeling the TME and stimulating both innate and adaptive immunity (Xiong et al. 2022).

## Future perspectives

Exosomes, as stable and biocompatible extracellular vesicles, play crucial roles in intercellular communication within the TME. They facilitate tumor progression, immune modulation, and drug resistance by transferring bioactive molecules such as proteins, lipids, and nucleic acids between cells. Being derived from tumor cells, immune cells, and stromal cells, they demonstrate dual roles in cancer, either promoting or inhibiting tumor growth based on their origin and cargo. This duality underscores their potential as both diagnostic biomarkers and therapeutic agents. Ongoing clinical trials in lung, breast, and colon cancer highlighting their potential are summarized in Table 1.

**Table 1:** Application of exosomes in ongoing clinical trials in lung, breast, and colon cancer (Data source: ClinicalTrials.gov: https://clinicaltrials.gov/ accessed in Dec 2024)

Clinical Trial Identifier	Phase	Designation	Therapy
NCT01294072	N/A	Colorectal Cancer	plant exosomes loaded with curcumin
NCT02977468	I	Triple Negative Breast Cancer	pembrolizumab
NCT04530890	N/A	Digestive or gynecological / breast cancer	chemotherapy, immunotherapy
NCT04499794	N/A	Advanced NSCLC Patients	ALK inhibitor
NCT05218759	N/A	Advanced Non-Small Cell Lung Carcinoma	anlotinib
NCT05798338	N/A	Breast Cancer	SiMoA diagnostic assay
NCT05831397	N/A	Breast Cancer	neoadjuvant chemotherapy, SiMoA - ELISA
NCT05854030	N/A	Lung Squamous Carcinoma	-
NCT06026735	N/A	Lung Cancer with CNS Metastasis	-
NCT06342401	N/A	Colorectal Cancer	ENCODE diagnostic test
NCT06342440	N/A	Advanced Adenomas, Colorectal Cancer	DENEB diagnostic test

Recent advancements highlight the promise of engineered exosomes in cancer therapy. These exosomes can be tailored to deliver specific therapeutic agents, such as small molecules, miRNAs, or immune modulators, enhancing targeting, stability, and efficacy. Beyond immunotherapy, exosomes are also explored as vehicles for chemotherapy delivery, reducing systemic toxicity and enhancing tumor targeting. The use of exosomal vaccines has gained attention due to their ability to induce antigen-specific immune responses and establish long-term immunological memory. Multimodal platforms, combining immune cell derived exosomes with anti-PD-1 antibodies, demonstrate significant immune activation and tumor suppression in preclinical models. Furthermore, exosome-based immunotherapy strategies, including dual targeting exosomes and hybrid chimeric exosomes, have shown efficacy in addressing the immunosuppressive challenges of the TME. Despite their potential, challenges remain, including non-specific tissue distribution, production scalability, and heterogeneity in natural exosomes. Advances in synthetic biology and nanotechnology aim to address these limitations, enabling the development of exosome mimetic systems that combine tumor specificity with enhanced therapeutic efficacy.

In conclusion, exosomes represent a transformative avenue in cancer therapy, offering novel strategies for drug delivery, immunotherapy, and vaccine development. Their integration into personalized medicine approaches holds promise for improving outcomes in solid tumors, although further clinical translation and standardization are required to realize their full potential.

## Data Availability

No datasets were generated or analysed during the current study.

## References

[CR1] Abbasi R, Nejati V, Rezaie J (2024) Exosomes biogenesis was increased in metformin-treated human ovary cancer cells; possibly to mediate resistance. Cancer Cell Int 24(1):137. 10.1186/s12935-024-03312-6 38627767 10.1186/s12935-024-03312-6 PMC11022479

[CR2] Ahmadi M, Hassanpour M, Rezaie J (2022) Engineered extracellular vesicles: a novel platform for cancer combination therapy and cancer immunotherapy. Life Sci 308:120935. 10.1016/j.lfs.2022.120935 36075472 10.1016/j.lfs.2022.120935

[CR3] Besse B, Charrier M, Lapierre V, Dansin E, Lantz O, Planchard D, Le Chevalier T, Livartoski A, Barlesi F, Laplanche A, Ploix S, Vimond N, Peguillet I, Théry C, Lacroix L, Zoernig I, Dhodapkar K, Dhodapkar M, Viaud S, Soria J-C, Reiners KS, Pogge Von Strandmann E, Vély F, Rusakiewicz S, Eggermont A, Pitt JM, Zitvogel L, Chaput N (2016) Dendritic cell-derived exosomes as maintenance immunotherapy after first line chemotherapy in NSCLC. OncoImmunology 5(4):e1071008. 10.1080/2162402X.2015.1071008 27141373 10.1080/2162402X.2015.1071008 PMC4839329

[CR4] Bianco P, Cao X, Frenette PS, Mao JJ, Robey PG, Simmons PJ, Wang C-Y (2013) The meaning, the sense and the significance: translating the science of mesenchymal stem cells into medicine. Nat Med 19(1):35–42. 10.1038/nm.3028 23296015 10.1038/nm.3028 PMC3998103

[CR5] Borges FT, Reis LA, Schor N (2013) Extracellular vesicles: structure, function, and potential clinical uses in renal diseases. Braz J Med Biol Res 46(10):824–830. 10.1590/1414-431X20132964 24141609 10.1590/1414-431X20132964 PMC3854311

[CR6] Campbell KS, Hasegawa J (2013) Natural killer cell biology: an update and future directions. J Allergy Clin Immunol 132(3):536–544. 10.1016/j.jaci.2013.07.006 23906377 10.1016/j.jaci.2013.07.006 PMC3775709

[CR7] Carvalho AL, Fonseca S, Miquel-Clopés A, Cross K, Kok K, Wegmann U, Gil‐Cardoso K, Bentley EG, Al Katy SHM, Coombes JL, Kipar A, Stentz R, Stewart JP, Carding SR (2019) Bioengineering commensal bacteria‐derived outer membrane vesicles for delivery of biologics to the gastrointestinal and respiratory tract. J Extracell Vesicles 8(1):1632100. 10.1080/20013078.2019.1632100 31275534 10.1080/20013078.2019.1632100 PMC6598475

[CR150] Chen Q, Bai H, Wu W, Huang G, Li Y, Wu M, Tang G, Ping Y (2020) Bioengineering bacterial vesicle-coated polymeric nanomedicine for enhanced cancer immunotherapy and metastasis prevention. Nano Lett 20 (1):11–21. 10.1021/acs.nanolett.9b02182 10.1021/acs.nanolett.9b02182 31858807

[CR8] Chen B, Sang Y, Song X, Zhang D, Wang L, Zhao W, Liang Y, Zhang N, Yang Q (2021) Exosomal miR-500a-5p derived from cancer-associated fibroblasts promotes breast cancer cell proliferation and metastasis through targeting USP28. Theranostics 11(8):3932–3947. 10.7150/thno.53412 33664871 10.7150/thno.53412 PMC7914354

[CR9] Chen X, Li J, Zhang R, Zhang Y, Wang X, Leung EL, Ma L, Wong VKW, Liu L, Neher E, Yu H (2022) Suppression of PD-L1 release from small extracellular vesicles promotes systemic anti‐tumor immunity by targeting ORAI1 calcium channels. J Extracell Vesicles 11(12):12279. 10.1002/jev2.12279 36482876 10.1002/jev2.12279 PMC9732629

[CR10] Cheng AN, Cheng L-C, Kuo C-L, Lo YK, Chou H-Y, Chen C-H, Wang Y-H, Chuang T-H, Cheng S-J, Lee AY-L (2020) Mitochondrial lon-induced mtDNA leakage contributes to PD-L1–mediated immunoescape via STING-IFN signaling and extracellular vesicles. J Immunother Cancer 8(2):e001372. 10.1136/jitc-2020-001372 33268351 10.1136/jitc-2020-001372 PMC7713199

[CR11] Chow A, Perica K, Klebanoff CA, Wolchok JD (2022) Clinical implications of T cell exhaustion for cancer immunotherapy. Nat Rev Clin Oncol 19(12):775–790. 10.1038/s41571-022-00689-z 36216928 10.1038/s41571-022-00689-z PMC10984554

[CR12] Cianciaruso C, Beltraminelli T, Duval F, Nassiri S, Hamelin R, Mozes A, Gallart-Ayala H, Ceada Torres G, Torchia B, Ries CH, Ivanisevic J, De Palma M (2019) Molecular profiling and functional analysis of macrophage-derived Tumor Extracellular vesicles. Cell Rep 27(10):3062–3080e11. 10.1016/j.celrep.2019.05.008 31167148 10.1016/j.celrep.2019.05.008 PMC6581796

[CR13] Cocucci E, Meldolesi J (2015) Ectosomes and exosomes: shedding the confusion between extracellular vesicles. Trends Cell Biol 25(6):364–372. 10.1016/j.tcb.2015.01.004 25683921 10.1016/j.tcb.2015.01.004

[CR14] Demaria O, Cornen S, Daëron M, Morel Y, Medzhitov R, Vivier E (2019) Publisher correction: harnessing innate immunity in cancer therapy. Nature 576(7785):E3–E3. 10.1038/s41586-019-1758-2 31745371 10.1038/s41586-019-1758-2

[CR15] DeNardo DG, Ruffell B (2019) Macrophages as regulators of tumour immunity and immunotherapy. Nat Rev Immunol 19(6):369–382. 10.1038/s41577-019-0127-6 30718830 10.1038/s41577-019-0127-6 PMC7339861

[CR16] Deng Z, Rong Y, Teng Y, Zhuang X, Samykutty A, Mu J, Zhang L, Cao P, Yan J, Miller D, Zhang H-G (2017) Exosomes miR-126a released from MDSC induced by DOX treatment promotes lung metastasis. Oncogene 36(5):639–651. 10.1038/onc.2016.229 27345402 10.1038/onc.2016.229 PMC5419051

[CR17] Elahi FM, Farwell DG, Nolta JA, Anderson JD (2020) Preclinical translation of exosomes derived from mesenchymal stem/stromal cells. Stem Cells 38(1):15–21. 10.1002/stem.3061 31381842 10.1002/stem.3061 PMC7004029

[CR18] Figueroa J, Phillips LM, Shahar T, Hossain A, Gumin J, Kim H, Bean AJ, Calin GA, Fueyo J, Walters ET, Kalluri R, Verhaak RG, Lang FF (2017) Exosomes from Glioma-Associated Mesenchymal stem cells increase the tumorigenicity of glioma stem-like cells via transfer of miR-1587. Cancer Res 77(21):5808–5819. 10.1158/0008-5472.CAN-16-2524 28855213 10.1158/0008-5472.CAN-16-2524 PMC5668150

[CR19] Fu W, Lei C, Liu S, Cui Y, Wang C, Qian K, Li T, Shen Y, Fan X, Lin F, Ding M, Pan M, Ye X, Yang Y, Hu S (2019) CAR exosomes derived from effector CAR-T cells have potent antitumour effects and low toxicity. Nat Commun 10(1):4355. 10.1038/s41467-019-12321-3 31554797 10.1038/s41467-019-12321-3 PMC6761190

[CR20] Guan L, Wu B, Li T, Beer LA, Sharma G, Li M, Lee CN, Liu S, Yang C, Huang L, Frederick DT, Boland GM, Shao G, Svitkina TM, Cai KQ, Chen F, Dong M-Q, Mills GB, Schuchter LM, Karakousis GC, Mitchell TC, Flaherty KT, Speicher DW, Chen YH, Herlyn M, Amaravadi RK, Xu X, Guo W (2022) HRS phosphorylation drives immunosuppressive exosome secretion and restricts CD8 + T-cell infiltration into tumors. Nat Commun 13(1):4078. 10.1038/s41467-022-31713-6 35835783 10.1038/s41467-022-31713-6 PMC9283393

[CR21] Hosseini R, Asef-Kabiri L, Yousefi H, Sarvnaz H, Salehi M, Akbari ME, Eskandari N (2021) The roles of tumor-derived exosomes in altered differentiation, maturation and function of dendritic cells. Mol Cancer 20(1):83. 10.1186/s12943-021-01376-w 34078376 10.1186/s12943-021-01376-w PMC8170799

[CR22] Hu JL, Wang W, Lan XL, Zeng ZC, Liang YS, Yan YR, Song FY, Wang FF, Zhu XH, Liao WJ, Liao WT, Ding YQ, Liang L (2019) CAFs secreted exosomes promote metastasis and chemotherapy resistance by enhancing cell stemness and epithelial-mesenchymal transition in colorectal cancer. Mol Cancer 18(1):91. 10.1186/s12943-019-1019-x 31064356 10.1186/s12943-019-1019-x PMC6503554

[CR23] Huang L, Rong Y, Tang X, Yi K, Qi P, Hou J, Liu W, He Y, Gao X, Yuan C, Wang F (2022) Engineered exosomes as an in situ DC-primed vaccine to boost antitumor immunity in breast cancer. Mol Cancer 21(1):45. 10.1186/s12943-022-01515-x 35148751 10.1186/s12943-022-01515-x PMC8831689

[CR24] Hyenne V, Apaydin A, Rodriguez D, Spiegelhalter C, Hoff-Yoessle S, Diem M, Tak S, Lefebvre O, Schwab Y, Goetz JG, Labouesse M (2015) RAL-1 controls multivesicular body biogenesis and exosome secretion. J Cell Biol 211(1):27–37. 10.1083/jcb.201504136 26459596 10.1083/jcb.201504136 PMC4602040

[CR25] Jiang M, Zhang W, Zhang R, Liu P, Ye Y, Yu W, Guo X, Yu J (2020) Cancer exosome-derived miR-9 and miR-181a promote the development of early-stage MDSCs via interfering with SOCS3 and PIAS3 respectively in breast cancer. Oncogene 39(24):4681–4694. 10.1038/s41388-020-1322-4 32398867 10.1038/s41388-020-1322-4

[CR26] Johnstone RM, Adam M, Hammond JR, Orr L, Turbide C (1987) Vesicle formation during reticulocyte maturation. Association of plasma membrane activities with released vesicles (exosomes). J Biol Chem 262(19):9412–9420. 10.1016/S0021-9258(18)48095-7 3597417

[CR27] Johnstone RM, Mathew A, Mason AB, Teng K (1991) Exosome formation during maturation of mammalian and avian reticulocytes: evidence that exosome release is a major route for externalization of obsolete membrane proteins. J Cell Physiol 147(1):27–36. 10.1002/jcp.1041470105 2037622 10.1002/jcp.1041470105

[CR28] Jong AY, Wu C, Li J, Sun J, Fabbri M, Wayne AS, Seeger RC (2017) Large-scale isolation and cytotoxicity of extracellular vesicles derived from activated human natural killer cells. J Extracell Vesicles 6(1):1294368. 10.1080/20013078.2017.1294368 28326171 10.1080/20013078.2017.1294368 PMC5345580

[CR29] Jung D, Shin S, Kang S, Jung I, Ryu S, Noh S, Choi S, Jeong J, Lee BY, Kim K, Kim CS, Yoon JH, Lee C, Bucher F, Kim Y, Im S, Song B, Yea K, Baek M (2022) Reprogramming of T cell-derived small extracellular vesicles using IL2 surface engineering induces potent anti‐cancer effects through miRNA delivery. J Extracell Vesicles 11(12):12287. 10.1002/jev2.12287 36447429 10.1002/jev2.12287 PMC9709340

[CR30] Keerthikumar S, Chisanga D, Ariyaratne D, Al Saffar H, Anand S, Zhao K, Samuel M, Pathan M, Jois M, Chilamkurti N, Gangoda L, Mathivanan S (2016) ExoCarta: a web-based compendium of Exosomal Cargo. J Mol Biol 428(4):688–692. 10.1016/j.jmb.2015.09.019 26434508 10.1016/j.jmb.2015.09.019 PMC4783248

[CR31] Laumont CM, Banville AC, Gilardi M, Hollern DP, Nelson BH (2022) Tumour-infiltrating B cells: immunological mechanisms, clinical impact and therapeutic opportunities. Nat Rev Cancer 22(7):414–430. 10.1038/s41568-022-00466-1 35393541 10.1038/s41568-022-00466-1 PMC9678336

[CR35] Li Y, Tao Y, Gao S, Li P, Zheng J, Zhang S, Liang J, Zhang Y (2018) Cancer-associated fibroblasts contribute to oral cancer cells proliferation and metastasis via exosome-mediated paracrine miR-34a-5p. EBioMedicine 36:209–220. 10.1016/j.ebiom.2018.09.006 30243489 10.1016/j.ebiom.2018.09.006 PMC6197737

[CR33] Li C, Teixeira AF, Zhu H-J, Ten Dijke P (2021) Cancer associated-fibroblast-derived exosomes in cancer progression. Mol Cancer 20(1):154. 10.1186/s12943-021-01463-y 34852849 10.1186/s12943-021-01463-y PMC8638446

[CR32] Li B, Fang T, Li Y, Xue T, Zhang Z, Li L, Meng F, Wang J, Hou L, Liang X, Zhang X, Gu Z (2022) Engineered T cell extracellular vesicles displaying PD-1 boost anti-tumor immunity. Nano Today 46:101606. 10.1016/j.nantod.2022.101606

[CR34] Li J, Li J, Peng Y, Du Y, Yang Z, Qi X (2023) Dendritic cell derived exosomes loaded neoantigens for personalized cancer immunotherapies. J Control Release 353:423–433. 10.1016/j.jconrel.2022.11.053 36470333 10.1016/j.jconrel.2022.11.053

[CR37] Liang Y, Zhang D, Li L, Xin T, Zhao Y, Ma R, Du J (2020) Exosomal microRNA-144 from bone marrow-derived mesenchymal stem cells inhibits the progression of non-small cell lung cancer by targeting CCNE1 and CCNE2. Stem Cell Res Ther 11(1):87. 10.1186/s13287-020-1580-7 32102682 10.1186/s13287-020-1580-7 PMC7045474

[CR36] Liang Y, Duan L, Lu J, Xia J (2021) Engineering exosomes for targeted drug delivery. Theranostics 11(7):3183–3195. 10.7150/thno.52570 33537081 10.7150/thno.52570 PMC7847680

[CR38] Lichterman JN, Reddy SM (2021) Mast cells: a New Frontier for Cancer Immunotherapy. Cells 10(6):1270. 10.3390/cells10061270 34063789 10.3390/cells10061270 PMC8223777

[CR39] Lin MJ, Svensson-Arvelund J, Lubitz GS, Marabelle A, Melero I, Brown BD, Brody JD (2022a) Cancer vaccines: the next immunotherapy frontier. Nat Cancer 3(8):911–926. 10.1038/s43018-022-00418-6 35999309 10.1038/s43018-022-00418-6

[CR40] Lin Z, Wu Y, Xu Y, Li G, Li Z, Liu T (2022b) Mesenchymal stem cell-derived exosomes in cancer therapy resistance: recent advances and therapeutic potential. Mol Cancer 21(1):179. 10.1186/s12943-022-01650-5 36100944 10.1186/s12943-022-01650-5 PMC9468526

[CR41] Liu C, Liu X, Xiang X, Pang X, Chen S, Zhang Y, Ren E, Zhang L, Liu X, Lv P, Wang X, Luo W, Xia N, Chen X, Liu G (2022a) A nanovaccine for antigen self-presentation and immunosuppression reversal as a personalized cancer immunotherapy strategy. Nat Nanotechnol 17(5):531–540. 10.1038/s41565-022-01098-0 35410368 10.1038/s41565-022-01098-0

[CR42] Liu C, Wang Y, Li L, He D, Chi J, Li Q, Wu Y, Zhao Y, Zhang S, Wang L, Fan Z, Liao Y (2022b) Engineered extracellular vesicles and their mimetics for cancer immunotherapy. J Control Release 349:679–698. 10.1016/j.jconrel.2022.05.062 35878728 10.1016/j.jconrel.2022.05.062

[CR43] Lu Z, Zuo B, Jing R, Gao X, Rao Q, Liu Z, Qi H, Guo H, Yin H (2017) Dendritic cell-derived exosomes elicit tumor regression in autochthonous hepatocellular carcinoma mouse models. J Hepatol 67(4):739–748. 10.1016/j.jhep.2017.05.019 28549917 10.1016/j.jhep.2017.05.019

[CR44] Lugini L, Cecchetti S, Huber V, Luciani F, Macchia G, Spadaro F, Paris L, Abalsamo L, Colone M, Molinari A, Podo F, Rivoltini L, Ramoni C, Fais S (2012) Immune Surveillance Properties of Human NK Cell-Derived exosomes. J Immunol 189(6):2833–2842. 10.4049/jimmunol.1101988 22904309 10.4049/jimmunol.1101988

[CR45] Luo H, Zhou Y, Zhang J, Zhang Y, Long S, Lin X, Yang A, Duan J, Yang N, Yang Z, Che Q, Yang Y, Guo T, Zi D, Ouyang W, Yang W, Zeng Z, Zhao X (2023) NK cell-derived exosomes enhance the anti-tumor effects against ovarian cancer by delivering cisplatin and reactivating NK cell functions. Front Immunol 13:1087689. 10.3389/fimmu.2022.1087689 36741396 10.3389/fimmu.2022.1087689 PMC9892755

[CR46] Maskalenko NA, Zhigarev D, Campbell KS (2022) Harnessing natural killer cells for cancer immunotherapy: dispatching the first responders. Nat Rev Drug Discov 21(8):559–577. 10.1038/s41573-022-00413-7 35314852 10.1038/s41573-022-00413-7 PMC10019065

[CR47] Meng F, Li L, Zhang Z, Lin Z, Zhang J, Song X, Xue T, Xing C, Liang X, Zhang X (2022) Biosynthetic Neoantigen displayed on bacteria derived vesicles elicit systemic antitumour immunity. J Extracell Vesicles 11(12):12289. 10.1002/jev2.12289 36468941 10.1002/jev2.12289 PMC9721206

[CR48] Morrissey SM, Zhang F, Ding C, Montoya-Durango DE, Hu X, Yang C, Wang Z, Yuan F, Fox M, Zhang H, Guo H, Tieri D, Kong M, Watson CT, Mitchell RA, Zhang X, McMasters KM, Huang J, Yan J (2021) Tumor-derived exosomes drive immunosuppressive macrophages in a pre-metastatic niche through glycolytic dominant metabolic reprogramming. Cell Metab 33(10):2040–2058e10. 10.1016/j.cmet.2021.09.002 34559989 10.1016/j.cmet.2021.09.002 PMC8506837

[CR49] Murray PJ (2017) Macrophage polarization. Annu Rev Physiol 79(1):541–566. 10.1146/annurev-physiol-022516-034339 27813830 10.1146/annurev-physiol-022516-034339

[CR50] Neviani P, Wise PM, Murtadha M, Liu CW, Wu C-H, Jong AY, Seeger RC, Fabbri M (2019) Natural killer–derived exosomal miR-186 inhibits Neuroblastoma Growth and Immune escape mechanisms. Cancer Res 79(6):1151–1164. 10.1158/0008-5472.CAN-18-0779 30541743 10.1158/0008-5472.CAN-18-0779 PMC6428417

[CR51] Ning T, Li J, He Y, Zhang H, Wang X, Deng T, Liu R, Li H, Bai M, Fan Q, Zhu K, Ying G, Ba Y (2021) Exosomal miR-208b related with oxaliplatin resistance promotes Treg expansion in colorectal cancer. Mol Ther 29(9):2723–2736. 10.1016/j.ymthe.2021.04.028 33905821 10.1016/j.ymthe.2021.04.028 PMC8417448

[CR52] Ostrowski M, Carmo NB, Krumeich S, Fanget I, Raposo G, Savina A, Moita CF, Schauer K, Hume AN, Freitas RP, Goud B, Benaroch P, Hacohen N, Fukuda M, Desnos C, Seabra MC, Darchen F, Amigorena S, Moita LF, Thery C (2010) Rab27a and Rab27b control different steps of the exosome secretion pathway. Nat Cell Biol 12(1):19–30. 10.1038/ncb2000 19966785 10.1038/ncb2000

[CR53] Pakravan K, Babashah S, Sadeghizadeh M, Mowla SJ, Mossahebi-Mohammadi M, Ataei F, Dana N, Javan M (2017) MicroRNA-100 shuttled by mesenchymal stem cell-derived exosomes suppresses in vitro angiogenesis through modulating the mTOR/HIF-1α/VEGF signaling axis in breast cancer cells. Cell Oncol (Dordr) 40(5):457–470. 10.1007/s13402-017-0335-7 28741069 10.1007/s13402-017-0335-7 PMC13001539

[CR54] Park K, Svennerholm K, Crescitelli R, Lässer C, Gribonika I, Lötvall J (2021) Synthetic bacterial vesicles combined with tumour extracellular vesicles as cancer immunotherapy. J Extracell Vesicles 10(9):e12120. 10.1002/jev2.12120 34262675 10.1002/jev2.12120 PMC8254025

[CR55] Qin F, Tang H, Zhang Y, Zhang Z, Huang P, Zhu J (2020) Bone marrow-derived mesenchymal stem cell‐derived exosomal microRNA‐208a promotes osteosarcoma cell proliferation, migration, and invasion. J Cell Physiol 235(5):4734–4745. 10.1002/jcp.29351 31637737 10.1002/jcp.29351

[CR56] Qu X, Liu B, Wang L, Liu L, Zhao W, Liu C, Ding J, Zhao S, Xu B, Yu H, Zhang X, Chai J (2023) Loss of cancer-associated fibroblast-derived exosomal DACT3-AS1 promotes malignant transformation and ferroptosis-mediated oxaliplatin resistance in gastric cancer. Drug Resist Updat 68:100936. 10.1016/j.drup.2023.100936 36764075 10.1016/j.drup.2023.100936

[CR57] Ren B, Cui M, Yang G, Wang H, Feng M, You L, Zhao Y (2018) Tumor microenvironment participates in metastasis of pancreatic cancer. Mol Cancer 17(1):108. 10.1186/s12943-018-0858-1 30060755 10.1186/s12943-018-0858-1 PMC6065152

[CR58] Shaban SA, Rezaie J, Nejati V (2022) Exosomes Derived from senescent endothelial cells contain distinct pro-angiogenic miRNAs and proteins. Cardiovasc Toxicol 22(6):592–601. 10.1007/s12012-022-09740-y 35441341 10.1007/s12012-022-09740-y

[CR59] Sharma P, Allison JP (2015) The future of immune checkpoint therapy. Science 348(6230):56–61. 10.1126/science.aaa8172 25838373 10.1126/science.aaa8172

[CR60] Shin S, Jung I, Jung D, Kim CS, Kang S-M, Ryu S, Choi S-J, Noh S, Jeong J, Lee BY, Park J-K, Shin J, Cho H, Heo J-I, Jeong Y, Choi SH, Lee SY, Baek M-C, Yea K (2022) Novel antitumor therapeutic strategy using CD4 + T cell-derived extracellular vesicles. Biomaterials 289:121765. 10.1016/j.biomaterials.2022.121765 36067566 10.1016/j.biomaterials.2022.121765

[CR61] So KA, Min KJ, Hong JH, Lee J-K (2015) Interleukin-6 expression by interactions between gynecologic cancer cells and human mesenchymal stem cells promotes epithelial-mesenchymal transition. Int J Oncol 47(4):1451–1459. 10.3892/ijo.2015.3122 26316317 10.3892/ijo.2015.3122

[CR62] Tay C, Tanaka A, Sakaguchi S (2023) Tumor-infiltrating regulatory T cells as targets of cancer immunotherapy. Cancer Cell 41(3):450–465. 10.1016/j.ccell.2023.02.014 36917950 10.1016/j.ccell.2023.02.014

[CR63] Teng F, Tian W-Y, Wang Y-M, Zhang Y-F, Guo F, Zhao J, Gao C, Xue F-X (2016) Cancer-associated fibroblasts promote the progression of endometrial cancer via the SDF-1/CXCR4 axis. J Hematol Oncol 9(1):8. 10.1186/s13045-015-0231-4 26851944 10.1186/s13045-015-0231-4 PMC4744391

[CR64] Théry C, Zitvogel L, Amigorena S (2002) Exosomes: composition, biogenesis and function. Nat Rev Immunol 2(8):569–579. 10.1038/nri855 12154376 10.1038/nri855

[CR65] Théry C, Ostrowski M, Segura E (2009) Membrane vesicles as conveyors of immune responses. Nat Rev Immunol 9(8):581–593. 10.1038/nri2567 19498381 10.1038/nri2567

[CR66] Turiello R, Capone M, Morretta E, Monti MC, Madonna G, Azzaro R, Del Gaudio P, Simeone E, Sorrentino A, Ascierto PA, Morello S (2022) Exosomal CD73 from serum of patients with melanoma suppresses lymphocyte functions and is associated with therapy resistance to anti-PD-1 agents. J Immunother Cancer 10(3):e004043. https://doi.org/10.1136/jitc-2021-004043 (2023) Correction: Exosomal CD73 from serum of patients with melanoma suppresses lymphocyte functions and is associated with therapy resistance to anti-PD-1 agents. J Immunother Cancer 11(7):e004043corr1. 10.1136/jitc-2021-004043corr1 10.1136/jitc-2021-004043 PMC8915288 35273100

[CR67] Veglia F, Sanseviero E, Gabrilovich DI (2021) Myeloid-derived suppressor cells in the era of increasing myeloid cell diversity. Nat Rev Immunol 21(8):485–498. 10.1038/s41577-020-00490-y 33526920 10.1038/s41577-020-00490-y PMC7849958

[CR68] Vitale I, Manic G, Coussens LM, Kroemer G, Galluzzi L (2019) Macrophages and metabolism in the Tumor Microenvironment. Cell Metab 30(1):36–50. 10.1016/j.cmet.2019.06.001 31269428 10.1016/j.cmet.2019.06.001

[CR69] Wakim LM, Bevan MJ (2011) Cross-dressed dendritic cells drive memory CD8 + T-cell activation after viral infection. Nature 471(7340):629–632. 10.1038/nature09863 21455179 10.1038/nature09863 PMC3423191

[CR70] Wang P, Wang H, Huang Q, Peng C, Yao L, Chen H, Qiu Z, Wu Y, Wang L, Chen W (2019a) Exosomes from M1-Polarized macrophages enhance Paclitaxel Antitumor activity by activating macrophages-mediated inflammation. Theranostics 9(6):1714–1727. 10.7150/thno.30716 31037133 10.7150/thno.30716 PMC6485189

[CR72] Wang X, Qin X, Yan M, Shi J, Xu Q, Li Z, Yang W, Zhang J, Chen W (2019b) Loss of exosomal miR-3188 in cancer-associated fibroblasts contributes to HNC progression. J Exp Clin Cancer Res 38(1):151. 10.1186/s13046-019-1144-9 30961650 10.1186/s13046-019-1144-9 PMC6454737

[CR74] Wang Y, Yin K, Tian J, Xia X, Ma J, Tang X, Xu H, Wang S (2019c) Granulocytic myeloid-derived suppressor cells promote the stemness of Colorectal Cancer cells through Exosomal S100A9. Adv Sci (Weinh) 6(18):1901278. 10.1002/advs.201901278 31559140 10.1002/advs.201901278 PMC6755519

[CR71] Wang S, Li F, Ye T, Wang J, Lyu C, Qing S, Ding Z, Gao X, Jia R, Yu D, Ren J, Wei W, Ma G (2021) Macrophage-tumor chimeric exosomes accumulate in lymph node and tumor to activate the immune response and the tumor microenvironment. Sci Transl Med 13(615):eabb6981. 10.1126/scitranslmed.abb6981 34644149 10.1126/scitranslmed.abb6981

[CR75] Wei Y, Tang X, Ren Y, Yang Y, Song F, Fu J, Liu S, Yu M, Chen J, Wang S, Zhang K, Tan Y, Han Z, Wei L, Zhang B, Cheng Z, Li L, Wang H (2021) An RNA–RNA crosstalk network involving HMGB1 and RICTOR facilitates hepatocellular carcinoma tumorigenesis by promoting glutamine metabolism and impedes immunotherapy by PD-L1 + exosomes activity. Signal Transduct Target Ther 6(1):421. 10.1038/s41392-021-00801-2 34916485 10.1038/s41392-021-00801-2 PMC8677721

[CR76] Welsh JA, Goberdhan DCI, O’Driscoll L, Buzas EI, Blenkiron C, Bussolati B, Cai H, Di Vizio D, Driedonks TAP, Erdbrügger U, Falcon-Perez JM, Fu Q, Hill AF, Lenassi M, Lim SK, Mahoney MG, Mohanty S, Möller A, Nieuwland R, Ochiya T, Sahoo S, Torrecilhas AC, Zheng L, Zijlstra A, Abuelreich S, Bagabas R, Bergese P, Bridges EM, Brucale M, Burger D, Carney RP, Cocucci E, Crescitelli R, Hanser E, Harris AL, Haughey NJ, Hendrix A, Ivanov AR, Jovanovic‐Talisman T, Kruh‐Garcia NA, Ku’ulei‐Lyn Faustino V, Kyburz D, Lässer C, Lennon KM, Lötvall J, Maddox AL, Martens‐Uzunova ES, Mizenko RR, Newman LA, Ridolfi A, Rohde E, Rojalin T, Rowland A, Saftics A, Sandau US, Saugstad JA, Shekari F, Swift S, Ter‐Ovanesyan D, Tosar JP, Useckaite Z, Valle F, Varga Z, Van Der Pol E, Van Herwijnen MJC, Wauben MHM, Wehman AM, Williams S, Zendrini A, Zimmerman AJ, MISEV Consortium, Théry C, Witwer KW (2024) Minimal information for studies of extracellular vesicles (MISEV2023): From basic to advanced approaches. J Extracell Vesicles 13(2):e12404. 10.1002/jev2.12404 (2024) Correction to Minimal information for studies of extracellular vesicles (MISEV2023): From basic to advanced approaches. J Extracell Vesicles 13(5):e12451. https://doi.org/10.1002/jev2.12451 10.1002/jev2.12404 PMC10850029 38326288

[CR77] Weng Z, Zhang B, Wu C, Yu F, Han B, Li B, Li L (2021) Therapeutic roles of mesenchymal stem cell-derived extracellular vesicles in cancer. J Hematol Oncol 14(1):136. 10.1186/s13045-021-01141-y 10.1186/s13045-021-01141-y PMC8414028 34479611

[CR78] Wolf P (1967) The Nature and significance of platelet products in human plasma. Br J Haematol 13(3):269–288. 10.1111/j.1365-2141.1967.tb08741.x 6025241 10.1111/j.1365-2141.1967.tb08741.x

[CR79] Wu C, Li J, Li L, Sun J, Fabbri M, Wayne AS, Seeger RC, Jong AY (2019) Extracellular vesicles derived from natural killer cells use multiple cytotoxic proteins and killing mechanisms to target cancer cells. J Extracell Vesicles 8(1):1588538. 10.1080/20013078.2019.1588538 30891164 10.1080/20013078.2019.1588538 PMC6419691

[CR80] Wu Q, Fu S, Xiao H, Du J, Cheng F, Wan S, Zhu H, Li D, Peng F, Ding X, Wang L (2023) Advances in Extracellular Vesicle Nanotechnology for Precision Theranostics. Adv Sci (Weinh) 10(3):2204814. 10.1002/advs.202204814 36373730 10.1002/advs.202204814 PMC9875626

[CR81] Xi L, Peng M, Liu S, Liu Y, Wan X, Hou Y, Qin Y, Yang L, Chen S, Zeng H, Teng Y, Cui X, Liu M (2021) Hypoxia-stimulated ATM activation regulates autophagy‐associated exosome release from cancer‐associated fibroblasts to promote cancer cell invasion. J Extracell Vesicles 10(11):e12146. 10.1002/jev2.12146 34545708 10.1002/jev2.12146 PMC8452512

[CR83] Xiao Y, Yu D (2021) Tumor microenvironment as a therapeutic target in cancer. Pharmacol Ther 221:107753. 10.1016/j.pharmthera.2020.107753 33259885 10.1016/j.pharmthera.2020.107753 PMC8084948

[CR82] Xiao H, Lässer C, Shelke GV, Wang J, Rådinger M, Lunavat TR, Malmhäll C, Lin LH, Li J, Li L, Lötvall J (2014) Mast cell exosomes promote lung adenocarcinoma cell proliferation– role of KIT-stem cell factor signaling. Cell Commun Signal 12(1):64. 10.1186/s12964-014-0064-8 25311367 10.1186/s12964-014-0064-8 PMC4206705

[CR85] Xie Y, Zhang X, Zhao T, Li W, Xiang J (2013) Natural CD8 + 25 + regulatory T cell-secreted exosomes capable of suppressing cytotoxic T lymphocyte-mediated immunity against B16 melanoma. Biochem Biophys Res Commun 438(1):152–155. 10.1016/j.bbrc.2013.07.044 23876314 10.1016/j.bbrc.2013.07.044

[CR84] Xie F, Zhou X, Su P, Li H, Tu Y, Du J, Pan C, Wei X, Zheng M, Jin K, Miao L, Wang C, Meng X, Van Dam H, Ten Dijke P, Zhang L, Zhou F (2022) Breast cancer cell-derived extracellular vesicles promote CD8 + T cell exhaustion via TGF-β type II receptor signaling. Nat Commun 13(1):4461. 10.1038/s41467-022-31250-2 35915084 10.1038/s41467-022-31250-2 PMC9343611

[CR86] Xiong X, Ke X, Wang L, Lin Y, Wang S, Yao Z, Li K, Luo Y, Liu F, Pan Y, Yeung SJ, Helfrich W, Zhang H (2022) Neoantigen-based cancer vaccination using chimeric RNA‐loaded dendritic cell‐derived extracellular vesicles. J Extracell Vesicles 11(8):e12243. 10.1002/jev2.12243 35927827 10.1002/jev2.12243 PMC9451527

[CR87] Yáñez-Mó M, Siljander PR, ‐M., Andreu Z, Bedina Zavec A, Borràs FE, Buzas EI, Buzas K, Casal E, Cappello F, Carvalho J, Colás E, Cordeiro‐da Silva A, Fais S, Falcon‐Perez JM, Ghobrial IM, Giebel B, Gimona M, Graner M, Gursel I, Gursel M, Heegaard NHH, Hendrix A, Kierulf P, Kokubun K, Kosanovic M, Kralj‐Iglic V, Krämer‐Albers E, Laitinen S, Lässer C, Lener T, Ligeti E, Linē A, Lipps G, Llorente A, Lötvall J, Manček‐Keber M, Marcilla A, Mittelbrunn M, Nazarenko I, Nolte‐‘t Hoen ENM, Nyman TA, O’Driscoll L, Olivan M, Oliveira C, Pállinger É, Del Portillo HA, Reventós J, Rigau M, Rohde E, Sammar M, Sánchez‐Madrid F, Santarém N, Schallmoser K, Stampe Ostenfeld M, Stoorvogel W, Stukelj R, Van Der Grein SG, Helena Vasconcelos M, Wauben MHM, De Wever O (2015) Biological properties of extracellular vesicles and their physiological functions. J Extracell Vesicles 4(1):27066. 10.3402/jev.v4.27066 25979354 10.3402/jev.v4.27066 PMC4433489

[CR88] Yu B, Zhang X, Li X (2014) Exosomes derived from mesenchymal stem cells. Int J Mol Sci 15(3):4142–4157. 10.3390/ijms15034142 24608926 10.3390/ijms15034142 PMC3975389

[CR89] Yue Y, Xu J, Li Y, Cheng K, Feng Q, Ma X, Ma N, Zhang T, Wang X, Zhao X, Nie G (2022) Antigen-bearing outer membrane vesicles as tumour vaccines produced in situ by ingested genetically engineered bacteria. Nat Biomed Eng 6(7):898–909. 10.1038/s41551-022-00886-2 35501399 10.1038/s41551-022-00886-2

[CR90] Zhan Y, Du J, Min Z, Ma L, Zhang W, Zhu W, Liu Y (2021) Carcinoma-associated fibroblasts derived exosomes modulate breast cancer cell stemness through exonic circHIF1A by mir-580-5p in hypoxic stress. Cell Death Discov 7(1):141. 10.1038/s41420-021-00506-z 34120145 10.1038/s41420-021-00506-z PMC8197761

[CR92] Zhang L, Yu D (2019b) Exosomes in cancer development, metastasis, and immunity. Biochim Biophys Acta Rev Cancer 1871(2):455–468. 10.1016/j.bbcan.2019.04.004 31047959 10.1016/j.bbcan.2019.04.004 PMC6542596

[CR91] Zhang F, Li R, Yang Y, Shi C, Shen Y, Lu C, Chen Y, Zhou W, Lin A, Yu L, Zhang W, Xue Z, Wang J, Cai Z (2019a) Specific decrease in B-Cell-derived extracellular vesicles enhances post-chemotherapeutic CD8 + T cell responses. Immunity 50(3):738–750e7. 10.1016/j.immuni.2019.01.010 30770248 10.1016/j.immuni.2019.01.010

[CR93] Zhang X, Zhang H, Gu J, Zhang J, Shi H, Qian H, Wang D, Xu W, Pan J, Santos HA (2021) Engineered Extracellular vesicles for Cancer Therapy. Adv Mater 33(14):2005709. 10.1002/adma.202005709 10.1002/adma.202005709 33644908

[CR94] Zhao S, Mi Y, Guan B, Zheng B, Wei P, Gu Y, Zhang Z, Cai S, Xu Y, Li X, He X, Zhong X, Li G, Chen Z, Li D (2020) Tumor-derived exosomal miR-934 induces macrophage M2 polarization to promote liver metastasis of colorectal cancer. J Hematol Oncol 13(1):156. 10.1186/s13045-020-00991-2 (2021a) Correction to: Tumor-derived exosomal miR-934 induces macrophage M2 polarization to promote liver metastasis of colorectal cancer. J Hematol Oncol 14(1):33. https://doi.org/10.1186/s13045-021-01042-0 10.1186/s13045-020-00991-2 PMC7678301 33213490

[CR95] Zhao X, Yuan C, Wangmo D, Subramanian S (2021) Tumor-secreted extracellular vesicles regulate T-Cell Costimulation and can be manipulated to induce tumor-specific T-Cell responses. Gastroenterology 161(2):560–574e11. 10.1053/j.gastro.2021.04.036 33895168 10.1053/j.gastro.2021.04.036

[CR96] Zheng P, Luo Q, Wang W, Li J, Wang T, Wang P, Chen L, Zhang P, Chen H, Liu Y, Dong P, Xie G, Ma Y, Jiang L, Yuan X, Shen L (2018) Tumor-associated macrophages-derived exosomes promote the migration of gastric cancer cells by transfer of functional Apolipoprotein E. Cell Death Dis 9(4):434. 10.1038/s41419-018-0465-5 29567987 10.1038/s41419-018-0465-5 PMC5864742

[CR97] Zhou W-J, Zhang J, Xie F, Wu J-N, Ye J-F, Wang J, Wu K, Li M-Q (2021) CD45RO - CD8 + T cell-derived exosomes restrict estrogen-driven endometrial cancer development via the ERβ/miR-765/PLP2/Notch axis. Theranostics 11(11):5330–5345. 10.7150/thno.58337 33859750 10.7150/thno.58337 PMC8039953

[CR99] Zhu W, Huang L, Li Y, Zhang X, Gu J, Yan Y, Xu X, Wang M, Qian H, Xu W (2012) Exosomes derived from human bone marrow mesenchymal stem cells promote tumor growth in vivo. Cancer Lett 315(1):28–37. 10.1016/j.canlet.2011.10.002 22055459 10.1016/j.canlet.2011.10.002

[CR98] Zhu L, Kalimuthu S, Oh JM, Gangadaran P, Baek SH, Jeong SY, Lee S-W, Lee J, Ahn B-C (2019) Enhancement of antitumor potency of extracellular vesicles derived from natural killer cells by IL-15 priming. Biomaterials 190–191:38–50. 10.1016/j.biomaterials.2018.10.034 30391801 10.1016/j.biomaterials.2018.10.034

